# Towards Retinoid Therapy for Alzheimer’s Disease

**DOI:** 10.2174/156720509788486581

**Published:** 2009-06

**Authors:** K Shudo, H Fukasawa, M Nakagomi, N Yamagata

**Affiliations:** 1 Research Foundation ITSUU Laboratory, Molecular and Functional Bioscience, Japan; 2 Institute of Medicinal Molecular Design, Inc., Molecular and Functional Bioscience, Japan

**Keywords:** Alzheimer’s disease, retinoid, retinoic acid receptors, inflammation, immunology, regulatory T cell, neurodegeneration.

## Abstract

Alzheimer’s disease(AD) is associated with a variety of pathophysiological features, including amyloid plaques, inflammation, immunological changes, cell death and regeneration processes, altered neurotransmission, and age-related changes. Retinoic acid receptors (RARs) and retinoids are relevant to all of these. Here we review the pathology, pharmacology, and biochemistry of AD in relation to RARs and retinoids, and we suggest that retinoids are candidate drugs for treatment of AD.

## TOWARDS RETINOID THERAPY FOR ALZHEIMER’S DISEASE

The management of Alzheimer’s disease (AD) remains a challenge, even as our knowledge and understanding of AD continue to grow at an unprecedented rate [1-3]. Research that bridges the gap between basic science and clinical application is extremely important for the development of new therapeutics. AD is a neurodegenerative disease, and has many pathological, biochemical and immunological features in common with Parkinson’s disease (PD), amyotrophic lateral sclerosis (ALS), multiple sclerosis (MS) and spinal code injury (SCI). This paper deals mainly with AD in relation to retinoic acid receptors (RARs: RARα, β and γ) and their ligands (retinoids), such as the endogenous RAR ligand all-*trans*-retinoic acid (RA), taking into account knowledge about PD, ALS and other neurodegenerative diseases. It is important to note that factors leading to the onset of these diseases are still poorly understood, and so there is a great deal of scope for novel therapeutic approaches. Recent findings indicate that the window of opportunity for enhancing or normalizing the growth of neuronal cells and promoting recovery from neurodegenerative diseases may be larger than previously thought.

AD is associated with a variety of pathophysiological features, including amyloid plaques, inflammation, immunological changes, cell death and regeneration processes, altered neurotransmission, and age-related changes. Here, we will review how retinoids could be involved in all these features of AD, together with the brief summary of their biology, pharmacology, and medicinal chemistry. Fig. (**1**) shows the overview of the various potential therapeutic targets by retinoids discussed in this paper.

## MEDICINAL CHEMISTRY OF RETINOIDS

Retinoids are analogues of retinoic acid, an active metabolite of vitamin A, and are specific modulators of cell proliferation, differentiation, and morphogenesis in vertebrates. There are many excellent reviews of the medicinal chemistry of retinoids [4-10]. The term retinoid is used for substances which may be defined as (1) vitamin A related compounds (including vitamin A (retinol) and its biological precursor carotenoids, (2) RA (vitamin A metabolite), which activates RARα, β, and γ, and synthetic analogs which bind with RARs with high affinity in an agonistic (similar biological activities to RA) or antagonistic manner, (3) compounds which activate RXRα, β, and γ, which are nuclear receptors different from RARs, (4) compounds which modify the activities of RA by influencing metabolism, biosynthesis or other pathways acting on co-factors, without binding to RARs or RXRs. We prefer to use the term retinoid in a strict sense, that is, compounds in category (2). It should be noted that the activity of vitamin A (retinol) is essentially due to RA generated by metabolism *in vivo*, except for the participation in vision via rhodopsin [11-13]. Normally, a low level of RA is present (~10^–9~10^ M), although a much larger amount (~10^-6 ^M) of vitamin A (retinol) is found in serum. The endogenous active retinoid is RA, which binds to and activates RARα, β, and γ. Isomeric 13-*cis*-retinoic acid may also be an endogenous retinoid, but is less abundant than RA. 9-*cis*-Retinoic acid is synthesized from, or in equilibrium with, RA, and it binds to and activates RARα, β, and γ, and in addition RXR α, β, and γ. Many synthetic analogs with a variety of structures have been prepared, and show different receptor selectivities and elicit different pharmacological effects from RA, including different adverse effects. Some act as antagonists which inhibit the activity of RA.

## BIOLOGICAL AND PHARMACOLOGICAL ACTIVITIES OF RETINOIDS

The biological importance of RARs has been thoroughly documented elsewhere [14]. A number of genes are regulated directly through the RAR or RXR- response elements through retinoic acid or retinoids binding to RAR of the RAR/RXR heterodimers, or indirectly, plausibly through the participation of the directly-regulated genes [15]. The biological or pharmacological activities of interest here are mostly covered in the above reviews [4-10]. An important practical clinical application of retinoids is in the treatment of acute promyelocytic leukemia (APL): treatment with RA results in complete remission in 90-95% of patients [16]. A synthetic retinoid, tamibarotene, works even on relapsed APL, which cannot be treated with RA [17]. Long-term maintenance treatments of APL patients using RA and tamibarotene are in progress. More recently a review of retinoids for cancer and metabolic disease was published [18]. Retinoids also inhibit angiogenesis [19]. Dermatological diseases, in particular psoriasis, are treated with RA, etretinate, and tamibarotene [20, 21]. Psoriasis is now regarded as an autoimmune disease [22]. Furthermore, retinoids is effective in the treatment of collagen-induced rheumatoid arthritis and other autoimmune models [23-25]. They are also useful to prevent atherosclerosis and restenosis of vascular vessels [26, 27]. Retinoids suppress the differentiation of preadipocytes to adipocytes [28], and promote alveolar regeneration in mammalian lungs [29]. Other observations suggest several clinical potential applications such as the treatment of diabetic retinopathy [30] and cataract [31]. Animal experiments using non-obese diabetic (NOD) mice suggested that retinoids may be effective to suppress type I diabetes and Schoegren’s syndrome [32]. Crohn’s disease is expected to be another treatment target [33, 34].

Retinoids appear to normalize many pathological states, and the clinical side effects presently reported are mostly not serious except for retinoic acid syndrome, which seems specific to APL pathogenesis. Teteratogenicity remains an issue during the organ formation period in pregnancy: absolute contraception is required until complete clearance. Genotoxicity has not been observed. Skin irritation often becomes a limitation in prolonged use for some retinoids; this may be caused by activation of the RARγ [35-38].

## ALZHEIMER’S PATHOPHYSIOLOGY

Current status of AD research are well summarized in a number of books, reviews, journals including this journal, and elsewhere such as web site reports [1-3, 39]. The major pathophysiology of AD is cerebral atrophy and neuronal loss, neuritic plaques and neurofibrillary tangles. Other important questions are being discussed such as “are inflammatory mechanisms actually causing damage in AD ?” [40] or “immune cells may fend off Alzheimer desease” [41]. Cell therapies of degenerated neural cells and tissues are of current interests, but relating to that, regeneration by retinoids looks actual and now well-understood [42, 43]. Age-related decline of cognition which is a serious feature of AD, could also be a target phenomenon of a retinoid [44, 45]. Several evidence for the relationships between the late onset Alzheimer’s disease and retinoid defective signaling are shown [46, 47]. Now AD and other neurodegenerative diseases may be likely the therapeutic targets of retinoid.

## AMYLOID-β HYPOTHESIS

AD is characterized by progressive memory deficits, cognitive impairment and personality changes due to neuronal cell death in the hippocampus and frontal cortex. The histopathological hallmark of AD is senile plaques, which consist of insoluble amyloid-β. Activated microglia and astrocytes (either proinflammatory or anti-inflammatory) surround the amyloid plaques, and appear to be associated with the lesions. Reduction of amyloid-β could be accomplished by inhibiting its production or aggregation, or promoting its degradation and removal, so active or passive immunization may have therapeutic potential. It has been shown that amyloid-β specific antibodies can clear amyloid assemblies and amyloid-β deposits in amyloid precursor protein (APP)-transgenic mice. Amyloid-β-specific antibodies have been extensively studied as a mediator of amyloid clearance [48].

APP is processed and fragmented by β-secretase and γ-secretase to generate amyloid-β. However, the nonamyloidogenic pathway of processing precursor proteins involves cleavage within the amyloid-β peptide sequence. The identification of a member of the disintegrin and metalloprotease family (ADAM10) as an α-secretase, whose expression can be regulated by retinoic acid, represents another therapeutic opportunity. Endogenous ADAM10 mRNA levels and the ADAM10 promoter activities were increased on RA treatment in neuroblastoma cells: thus, retinoic acid works as an activator of the α-secretase [49-51].

Dietary deficiency of vitamin A disrupts the retinoid signaling pathway in adult rats, leading to deposition of amyloid-β in the cerebral blood vessels via down-regulation of RAR-α in the forebrain neurons and loss of choline acetyltransferase (ChAT) expression, and these changes were reversed by administration of retinoic acid [52, 53]. Pathological samples from AD patients showed a similar RARα deficit and deposition of amyloid-β in the surviving neurons [52].

## NEUROINFLAMMATION

For many years, the central nervous system was considered to be immunologically privileged, being neither susceptible to, nor contributing to, inflammation. However, the Neuroinflammation Working Group suggested a relationship between inflammation and AD [40]. At least 600 reports indicate that the brain is by no means an immunologically isolated organ, and may have unique immunologic properties. Microglial-cell activation and migration, participation of astrocytes, and participation of various cytokines in AD have been confirmed. Microglia secrete proteolytic enzymes that degrade amyloid-β, and play a neuroprotective role in AD. One of the chemokine receptors, CCR2 deficiency impairs microglial accumulation and accelerates the progression of AD-like disease in a model mouse, Tg2576 [41, 54]. More recently, Beers *et al.* showed CD4+ T cells, which can be recruited by MCP-1/CCR2 signaling, provides supportive neuroprotection by modulating the trophic/cytotoxic balance of glia in ALS mouse mode [55].

Epidemiological studies indicate a reduced prevalence of AD among chronic users of nonsteroidal anti-inflamatory drugs, and clinical trials suggested a beneficial effect of some cycloxygenase (cox) inhibitors, although the effect is not related to the cox-inhibitory activity. Synthetic glucocorticoids are potent ant-inflammatory agents that act by antagonizing AP1 and NF-kB promoter elements that regulate the transcription of inflammatory molecules. They are useful for the treatment of inflammation, but various biochemical, systemic and behavioral side effects and other contraindications exist for use in AD. In their review [40], Akiyama *et al.* concluded that “no more than nonsteroidal anti-inflammatory drugs (NSAIDs) cure arthritis will anti-inflammatory drugs cure AD. However, if AD neuroinflammation is approached with realistic expectations and rational drug design, AD patients should significantly benefit from anti-inflammatory treatment”. Suppression of inflammation in the brain is important in the treatment of many neurodegenerative diseases.

Other aspects of inflammation and immunology of neurodegenerative diseases have also recently been reviewed [56-58]. Cytokines and chemokines, such as IL1α, IL1β, IL6, IL4, IL10, IL13 and MCP-1 are also important in AD. Though the action of IL6 generally depends on cellular and environmental conditions, unlike that of IL1β, which predominantly has a proinflammatory effect, the cytokine may generally exert a damaging proinflammatory effect in the central nervous system, although it is present in normal brain tissues [59]. IL6 dysregulation is involved in many age-related diseases, including autoimmune diseases. Chronic inflammation and astrocytosis are histopathological hallmarks of AD patients, and astrocytes and microglia produce IL6 in response to amyloid-β induced injury, thereby further promoting plaque formation. The induction of IL6 mRNA in the hippocampus and cortex of APPsw transgenic mice Tg2576 may be crucial in the early onset of AD [60]. The association of –174G/C and –572G/C mutations of IL6 promoters with AD has been discussed, but remains contentious [61, 62].

Since retinoids strongly suppress the production of IL6 [63, 64], the suppression of IL6 by retinoids may be beneficial for the treatment of AD. Retinoids also inhibit LPS- or amyloid-β-induced TNF-α production, and expression of inducible NO synthase (iNOS) in activated microglia; these effects may be mediated via the inhibition of NF-κB nuclear translocation. Retinoids inhibit many aspects of microglial activation [65, 66]. Thus, retinoids seem to have considerable potential from the standpoint of suppression of inflammation in neurodegenerative diseases.

## AUTOIMMUNE FEATURES

Immune system alterations during aging are complex and pleiotropic. Generally, changes of the T cell component are age-dependent. It is always difficult to determine whether changes are associated with aging itself or a result (or a cause) of disease. Although immunological studies of the brain have been relatively limited because of the supposed immune privilege of the brain, the role of immunology cannot be neglected in AD (or PD), as well as in the representative autoimmune neurodegenerative disease, MS. It is, however, interesting that up-regulation of major histocompatibility complex (MHC) class I and II on glial and neural cells occurs in AD, in addition to production of inflammatory cytokines and limited T cell infiltration [40, 67], and the generation of autoreactive antibodies and T cells against amyloid-β [68] . Similar findings were reported in PD and ALS [69-72]. Thus, there is a possibility that an appropriate modulation of autoimmune response may prevent disease development. In other words, changes in the activity or population of T cells may influence the progression of AD, PD and other such diseases.

Recently, IL-17-producing helper T cell subset, Th17 is at the center of attention in autoimmune research [73, 74]. The importance of Th17 cells in central nervous system inflammation in MS patients and experimental autoimmune myasthenia gravis in C57BL/6 mice has been shown [75, 76]. IL17 and IL22 receptors are expressed on blood-brain-barrier (BBB) endothelial cells in MS lesions, and these cytokines disrupt BBB tight junctions *in vivo* and *in vitro*. Thus, Th17 lymphocytes transmigrate efficiently across the BBB, killing human neurons and causing central nervous system inflammation through CD4+ lymphocyte recruitment. It was also shown that the BBB induces differentiation of migrating monocytes into Th17-polarizing dendritic cells [77]. The abundance of dendritic cells promotes secretion of IL12p70, TGFβ and IL6, as well as the proliferation and expansion of distinct populations of IFNγ-secreting Th1, and IL-17-secreting CD4+ Th17 lymphocytes. Such cells are closely associated with microvascular BBB endothelial cells within acute MS lesions. It has also been shown that impairment of the function of tight junctions facilitates the invasion of inflammatory cells prior to motor neuronal degeneration in ALS-causing SOD1 mutant mice [78]. Similar BBB disruption may be involved in the early onset of AD, as well as other neurodegenerative disorders. The suppression of Th17 or the restoration or maintenance of the function of tight junctions has a favorable effect, slowing the progression of autoimmune diseases. It is noteworthy that experimental autoimmune encephalomyelitis (EAE) mice or rats, an animal model of MS, has been successfully treated with retinoid [79-81]. Psoriasis, which is an important clinical target of retinoid, is now regarded as an autoimmune disease [22].

## T CELL DIFFERENTIATION

If T cells play a significant role in neurodegenerative diseases, their differentiation status must be important (Fig. **2**). For two decades, it has been known that the CD4+ cells among the naïve T cells can differentiate into two types of helper T cells, Th1 cells, which express the transcription factor T-bet (activated by IFNγ), and Th2 cells, which express the transcription factor GATA3 (activated by IL4). In this classical Th1/Th2 paradigm, which is revised by recent emergence of Th17, Th1 activation appears particularly important in tissue-specific autoimmunity such as EAE, and Th2 seems to be important in more systemic autoimmunity such as systemic lupas erythematosus (SLE) and allergic disease, though the key point is likely to be the balance between Th1 and Th2. It is noteworthy that the Th1 pathway is suppressed by RA (or retinoids), and the Th2 pathway is somewhat activated [82]. Retinoids are often beneficial for the treatment of autoimmune disease depending on the characteristics of the retinoid and the Th1/Th2 ratio of the disease [83]. A striking recent finding is that CD4+ naïve T cells, independently of Th1- and Th2-cell development, can differentiate to another helper T cell subset, Th17, which produces IL17 and expresses transcription factor retinoid-related orphan receptor γt (RORγt) [84]. Th17 cells are implicated in the pathogenesis of various autoimmune conditions, as mentioned in previous section, and supposed to be the major cause of rheumatoid arthritis, Crohn’s disease, psoriasis, MS and other diseases.

These effector T cells, Th1, Th2, and Th17 are suppressed by another CD4+ T cell subset of reguratory Tcells, Treg [85, 86]. A specific marker for Treg is the Foxp3 [87, 88]. The role of Treg cells in self-tolerance, autoimmune diseases and anti-tumor immunity has been actively studied in recent years. The decrease of Treg function may cause or progress breakdown of immune homeostasis. For example, the involvement of Treg has been demonstrated in MS [89], myasthenia gravis [90], and human T-lymphotropic virus 1 (HTLV-I) associated neuroimmunological disease [91]. Therapies for activation or proliferation of Treg may be useful for these autoimmune diseases.

This is also the case in the neurodegenerative diseases. Rosenkranz et al reported the involvement of Treg in AD and PD [92]. Analysis of Treg from AD and PD patients, as well as non-affected individuals, revealed that the frequency of Treg (CD4+ Foxp3+) increases with age and the increase is accompanied with intensified suppressive activity of Treg in patients. This may reflect a biological homeostatic response to the disease. Rosenkranz et al speculated that changes in Treg might affect disease-related immune mechanisms, since it has been hypothesized that neuroinflammation may be a response that is aimed at blocking disease development. The important role of Treg in PD was experimentally demonstrated in a MTPT (N-methyl-4-phenyl-1,2,3,6-tetrahydropyridine)-treated mouse model: Treg that highly expressed Foxp3 and IL10 was found to mediate neuroprotection through suppression of microglial activation [93].

**Fig. (1). Retinoid Dramatis Personae (Characters Involved in Retinoid Therapy).**
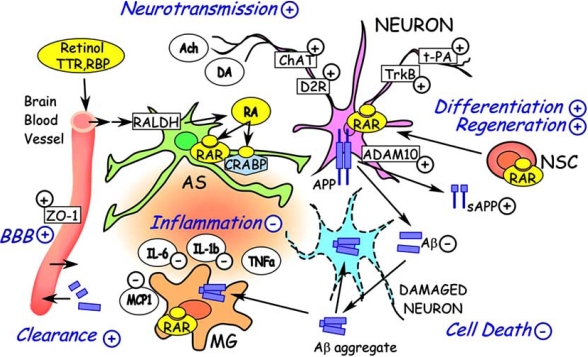
Neuron, dameged neuron, astrocyte (AS), microglia (MG), blood brain barrier (BBB), and other cellular members participate in the pathology of Alzheimer's disease. Retinol bound to retinol-binding protein (RBP) and TTR is transported from blood into brain. Retinol uptaked by brain cells is oxidised into retinal, then converterd into retinoic acid (RA). Cellular RA level is also regulated by cellular retinoic acid-binding proteins (CRABPs). By increase of α-secretase activity through ADAM10 induction and by other pathaways, retinoids decrease amyloid-β (Aβ) deposition from APP. Inflammatory response triggered by amyloid-β and other causes such as xenobiotics or chemicals is drived by inflammatory or proinflammatory cytokines and chemokines (IL-1b, IL-6, TNFa, MCP1 and others). Their production is suppressed by retinoids through RARs expressed in astrocyte and microglia. Cell death may be inhibited by the decrease of Aβ and suppression of inflammation. In regeneration from neural stem cells (NSC), retinoids have critical roles for their differentiation. Neurotransmission by Ach, DA and other molecules are affected by ChAT, dopamine D2 receptor (D2R), TrkB and t-PA which are transcriptionally regulated directly or indirectly by retinoid, influencing memory and learning. In addition, autoimmune pathways are regulated by T cells (Fig. **2** vide infra). In the scheme, + and - mean upregulation and down-regulation of the events or activities, by retinoids, respectively.

**Fig. (2). Multiple actions of retinoid in T cell differentiation.**
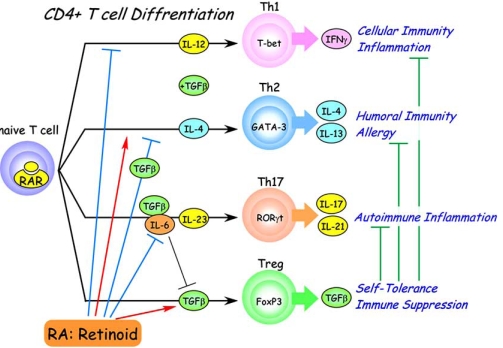
Naive CD4-positive T cells differntiate into effector T cells (Th1, Th2, Th17) and regulatory T cells (Treg) in peripheral lymphoid tissues. Th1 cells whose differentiation is dependent on IL-12 and suppressed by retinoids mediate cellular immunity and physiologocal (and pathological) inflammation. Th2 cells mediate humoral immunity and allegy. Their differentiation is dependent on IL-4 and enhanced by retinods, but suppressed by coexistence of retinoids and TGFβ. IL-17-producing Th17 cells mediate pathological chronic inflammation or autoimmune inflammation. Th17 diffrentiation is induced by coexistence of IL-6 and TGFβ, and maintained by IL-23. By contrast, differentiation of Treg cells is induced by TGFβ alone and suppressed by IL-6. Treg cells suppress effector T cell activities and and thereby maintain immune system homeostasis and self-tolerance. Retinoids strongly enhance TGFβ-induced Treg diffenentiation and suppress Th17 differentiation even in the presence of IL-6.

In these respects, it is striking that CD4+ Foxp3+ T cell development from naïve CD4+ T cells is strongly induced by co-treatment with TGFβ and retinoid, including RA and synthetic retinoids such as Am580, and is suppressed by the retinoid antagonist LE540 [94-102] (Fig. **1**). It is claimed that Treg is induced at the expense of IL17-secreting T cells by retinoic acid via RARα [102]. However, it is not yet clear whether Th17 cells are convertible to Treg, and also whether or not Foxp3-positive cells induced by RA and retinoids are true Treg. Much remains unclear about the roles of the various T cell subsets, but the importance of retinoids in T cell differentiation seems now clear. And, there is a proposal as to whether oral tolerance is all due to RA [103], where the author expects the use of Treg cells in the clinic and new RAR agonists other than RA with less side effects for *in vivo* uses.

## INFECTION

Infection could be a cause for breakdown of immune system and trigger autoimmune diseases. There is a hypothesis that AD is an autoimmune disease caused by an infection, but others have doubted a role of viral or other infection [104-106]. The role of infection with herpes simplex, spirochetes, and chlamydophila in AD has recently been reviewed [67, 107-109]. A role of infection would be consistent with a recent report that a group of ALS patients showed an extremely high frequency of systemic mycoplasmal infections [110].

Various routes of infection, such as gut, nasal, skin and lung, involving viruses, bacteria, specific proteins and chemical substances have been discussed. The olfactory vector hypothesis is that xenobiotics, including viruses and toxins, immunologically pull the trigger leading to neurodegeneration [106, 111]. This hypothesis is supported by the finding that olfactory dysfunction is a risk factor for PD and AD [111-113]. The normal differentiation and regeneration of olfactory-related cells are also regulated by retinoic acid, and vitamin A therapy in animals with olfactory system damage can accelerate functional recovery through RARs and retinoid X receptors (RXRs): retinoic acid supports the integrity of the olfactory system throughout life [114, 115]. The maintenance of olfactory function by retinoid may be preventive for the disease. Pathogens may elicit an autoimmune response without persistence of the initiating agent, environmental factors and nutrition being critical determinants of the onset.

## REGENERATION OF NEURAL CELLS

A possible approach to neurodegeneration is neural stem cell therapy. It is now well established that cells in the central nervous system can regenerate under certain conditions. Advances in techniques to isolate and manipulate neural stem cells offer new scope for functional recovery after central nervous system injuries.

Patterning into forebrain, midbrain, hindbrain, and spinal cord involves a complex series of morphogenic movements. Further differentiation is also strictly regulated: for example, stem cells at the CA1 region of hippocampus differentiate into the neural cells of CA1. It is also important that regeneration stops at the appropriate point. In many cases of regeneration and differentiation in neural injuries, retinoic acid is involved, or retinal dehydrogenases (RALDH) are produced. For example, inflammation reactions caused by spinal cord contusion in rats were followed by an increase of RALDH2 enzyme activity and local synthesis of RA [116-118]. Intracellular translocation of RARα (and RXRs) into the nuclei of activated macrophages, surviving neurons and astrocytes near the lesion site has been reported [119]. The localization of retinoid receptors in Schwann cells correlated with inflammatory transduction pathways of IL1β, IL6 and TNFα [120].

The activities of RA in in-vitro differentiation, as well as in the development of vertebrates, have been known for a long time. RA can induce the complete regeneration of organs that cannot normally regenerate, such as mammalian lung and retina, by respecifying positional information [42, 121]. Murine F9 teratocarcinoma stem cells have been widely used as a model for cellular differentiation and RA signaling during embryonic development [122]. Induction of neural differentiation by retinoid has been shown in several embryonal carcinoma cell lines [123-125]. Many studies on pattern formation have confirmed the importance of retinoids in spinal cord and brain development [126]. These findings prompted detailed analyses of RARs in brain [127], and extensive studies of RAR signaling pathways in neurological diseases [47]. The results suggest that RA or more generally retinoids are potential therapeutic agents for the treatment of neurodegenerative diseases, promoting tissue regeneration. Studies are advanced in the case of acute phase treatment of SCI. Mey analyzed the distribution and re-distribution of RARs after SCI [119]. Maden showed the importance of RARs in SCI, and found that RARβ gene introduction and the coexistence of a RARβ ligand (rather than RARγ) caused neurite growth and improved the recovery of animals after trauma [126, 128, 129]. Takenaga recently observed similar therapeutic effect in physically injured rats treated with the synthetic retinoid tamibarotene [17] which is RARα and RARβ specific (unpublished). Since the physical contusion is accompanied by inflammation and activation of microglia and macrophages, the therapeutic effect may not be simply explained, but at least re-generation and differentiation of neurite cells are promoted by retinoid in an adjuvant fashion.

Of course, other nuclear receptors (and their ligands) in addition to RARs participate, particularly RXRs, RORs, liver X receptor (LXR), orphan nuclear receptor NR4A2 (Nurr1) [130], and peroxisome proliferators-activated receptors (PPARs) [131]. The expression profile and functional role of nuclear receptors in such retinoid signaling have been profiled in human SCI, ALS, AD and PD [132].

## NEUROTRANSMISSION

Particular hallmarks observed in AD are the changes of cholinergic neurotransmission, which cause the decrease of acetylcholine (ACh). It was reported that vitamin A deficient rats with cognitive deficits showed impaired scopolamine-evoked release of ACh owing to blockade of the presynaptic acetylcholine autoreceptor. The impaired release of ACh is probably caused by lower production of ChAT, as well as neural cell death, mediated by RA and the gene promoter [133, 134].

An increase of ChAT seems to be beneficial, and it has been suggested that the vascular ACh transporter is also regulated by RA [135]. Retinoid rescued scopolamine-induced memory deficits in a passive avoidance paradigm [136]. This result could not be satisfactorily explained, but may support the participation of ACh and ChAT in the scopolamine-related memory deficit. In addition, retinoids also regulate the expression of tyrosine hydroxylase, dopamine β–hydroxylase and the dopamine D2 receptor. RA directly regulates D2 receptor expression via interaction with a RA-response element (RARE) at the promoter [137]. Such memory acquisition may not be explained simply by the participation of neurotransmitters.

## LEARNING AND MEMORY

The physiological role of retinoids in hippocampal function of adult brain has been reviewed by Lane and Bailey [138]. The enzymes that synthesize RA and RALDH protein are restricted to the meninges surrounding the hippocampus in adult brain. The presence of cellular retinol binding protein 1 (CRBP1) and cellular retinoic acid binding protein 1 (CRABP1) in the dendritic layers of the hippocampal formation and dentate gyrus has been demonstrated. RARα and RARγ, rather than RAR-β, are highly expressed in hippocampal CA1, CA2 and CA3 regions. RXRs are also expressed. These findings suggest that RA (and therefore retinoids) may play a central role in memory and spatial learning. Impaired long-term potentiation (LTP) and long-term depression (LTD) have been demonstrated in mice lacking RAR β alone, or RAR β and RXR γ [139]. Loss of synaptic plasticity is observed in vitamin A deficient mice [44] or aged mice [140, 141]. The reductions of LTD and LTP are partially reversed by RA, although the precise involvement of specific genes in the regulation of synaptic plasticity has not been elucidated.

Etchamendy *et al.* [140] demonstrated that pharmacological activation of retinoid signaling by short-term RA administration to aged mice restored their hippocampal retinoid target-gene hypoexpression, as well as their long-term declarative memory (LTDM) and hippocampal LTP deficit. Further, hippocampal retinoid receptor RARβ/RXRγ signaling is critically implicated in the cellular mechanism sustaining LTDM, as well as short-term working memory systems [45]. LTP-related genes, tyrosine kinase B (TrkB) and tissue plasminogen activator (t-PA), are reported to be the RAR-target genes and induced in neurobalstoma cells by treatment of retinoic acid [142-144]. TrkB is the receptor for brain-derived neurotrophic factor (BDNF), which is a key regulator for protein synthesis-dependent LTP and long-term memory and whose maturation is dependent on t-PA-mediated cleavage of proBDNF [145]. RARβ / RXRγ knockout mice behaved similarly to the aged mice and it was shown that RARβ and expression of neuromodulin (Gap43) gene, a plasticity related retinoid-target gene, are important. This paper also reviewed the literature on this subject.

Another interesting observation was the involvement of transthyretin (TTR), which was identified as a key protein by microarray analysis of genes associated with age-related memory deficits [146]. TTR is a serum and cerebrospinal fluid carrier of the thyroid hormone, thyroxine. TTR is also a carrier of retinol from liver storage to target tissues in association with retinol binding protein (RBP) [147]. TTR has been reported to be the major amyloid-β binding protein in cerebrospinal fluid. Age-related memory deficits occurred in TTR-/- mice in the water maze work task, and surprisingly, the deficits were improved by uptake of RA [146]. It has been shown that AD patients have lower levels of TTR [148]. TTR and possibly a similar serum protein, insulin-like growth factor I (IGF-I) regulate brain amyloid-β levels [149, 150]. Decreased levels of TTR may affect retinoid homeostasis, which is required to regulate neurogenesis and differentiation.

The appearance of new neurons in the hippocampus and cortex during adulthood seems to be linked to memory and learning [151]. Interestingly, in AD patients, an increased number of newly generated cells was observed in the granule cell layer, as well as the CA1 region of Ammon’s horn, where extensive cell death occurs during the disease [152]. Retinoids may have important roles in such neurogenesis.

## CONCLUSION

Much research in AD is still aimed at elucidating basic pathomechanisms, although over seventy-five compounds are in clinical development for treatment of AD [39].

Retinoids are required for the maintenance of the immune systems, and are very potent immunomodulators. They suppressively regulate various autoimmune disease states, and being different from simple immunosuppresants, work physiologically even when applied at high pharmacological concentrations.

Retinoids are essential in the regeneration of neural cells and other tissues. Development of retinoids that are highly selective for individual RARs may contribute to the treatment of AD and other neurodegenerative diseases. Recently there came out a review emphasizing significant roles of retinoids for treatment of neurodegenerative diseases such as ALS, AD and schizophrenia [153]. We did not cover RXRs and RXR ligands here, though these may have a role in increasing the selectivity of retinoids, and they seem to have important roles in mental activities such as sleep regulation, reward-related behaviors [154-158].

Retinoids were suggested long ago to have potential for the therapy of various proliferative diseases [159]. Some applications have been realized, and our task now is to extend the range of applications to neurodegenerative diseases, including AD.

## ABBREVIATIONS

AD: = Alzheimer’s disease
PD: = Parkinson’s disease
ALS: = Amyotrophic lateral sclerosis
MS: = Multiple sclerosis
SCI: = Spinal code injury
RARs: = Retinoic acid receptors
RA: = all-*trans*-retinoic acid
APP: = Amyloid precursor protein
ChAT: = Choline acetyltransferase
Treg: = Regulatory T cells
EAE: = Experimental autoimmune encephalomyelitis
RXRs: = Retinoid X receptors
RORs: = Retinoid-related orphan receptors
RALDH: = Retinal dehydrogenase
Ach: = Acetylcholine
CRBP1: = Cellular retinol binding protein 1
CRABP1: = Cellular retinoic acid binding protein 1
TTR: = Transthyretin
APL: = Acute promyelocytic leukemia

## NOTE ADDED IN PROOFS

Ref. [160]
